# Climate indicators for Austria since 1961 at 1 km resolution

**DOI:** 10.1038/s41597-026-06834-y

**Published:** 2026-02-18

**Authors:** Sebastian Lehner, Matthias Schlögl

**Affiliations:** 1 Department for Climate Impact Research, GeoSphere Austria, Hohe Warte 38, Vienna, 1190 Austria; 2 Department of Meteorology and Geophysics, University of Vienna, Josef-Holaubek-Platz 2, Vienna, 1090 Austria; 3 Institute of Mountain Risk Engineering, BOKU University, Peter-Jordan Straße 82, Vienna, 1190 Austria

## Abstract

Climate indicators are essential for monitoring ongoing climate change, supporting climate impact research, conducting spatial hot spot analyses and assessing attribution questions. These efforts rely on high-quality, reliable datasets that adhere to FAIR data principles. We present a curated dataset of 117 climate indicators for Austria, covering the period from 1961 onward at a 1-km spatial resolution. The dataset includes climate indicators related to temperature, precipitation, radiation, snow, runoff and humidity, with spatial (area means) and temporal (climatological reference period means) aggregations to enable rapid climate impact analysis. The workflow used to compute these indices is supported by a careful technical validation procedure and is designed to ingest diverse climate datasets, enabling the creation of climate indices beyond the scope presented here. Both the dataset and the workflow thus offer a robust, flexible and user-friendly resource for advancing climate research and supporting informed decision-making.

## Background & Summary

Climate change is one of the most pressing issues that affect humanity in the 21st century^1^. Addressing this global issue requires access to high-quality, reliable data to facilitate robust analyses of the climate system, assess emerging impacts, and inform evidence-based decision making^2^. Climate indicators, which distill raw meteorological data into useful impact-oriented metrics, are an important concept to isolate robust and significant signals from the noise of daily and internal climate variability^3^. However, a notable gap persists in the availability of accessible, user-friendly, high-resolution and well-curated climate indicator datasets. To bridge this gap, we introduce a comprehensive and quality-controlled dataset encompassing 117 climate indicators, covering common meteorological variables (temperature, precipitation, radiation, snow, runoff and humidity).

This dataset is intended to serve as a comprehensive catalog of climate indicators across various temporal scales and meteorological parameters, designed to accelerate climate (impact) analyses. Climate indicators are provided for seasonal and annual aggregation periods, as well as temporal averages across two adjacent climatological standard normals covering periods of 30 years each. Following the World Meteorological Organization (WMO) recommendation^4^, the reference period 1961–1990 is included in order to provide consistent comparisons with historical analyses, alongside the most recent reference period, 1991–2020, as adopted by the Copernicus Climate Change Service (C3S)^5^.

To facilitate rapid assessments across time, we provide spatial averages of all climate indicators, thereby simplifying the analysis of time series trends, shifts in governing patterns and extreme events. The dataset is built upon quality-controlled input data, and all climate indicators undergo thorough technical validation to ensure reliable outputs.

An earlier version of this dataset has already been used in^6^ to investigate physioclimatic regions of Austria. In that study, the climate indicators served as a basis for dimensionality reduction and high-dimensional clustering to delineate spatial clusters representing regional physioclimatic characteristics. Future research plans include a spatial hot spot analysis of climate change patterns and a comprehensive correlation analysis of climate indicator signals to identify the most representative indicators to transport information on relevant patterns. These efforts aim to (1) address challenges posed by multicollinearity, particularly in data-driven climate impact research applications that involve statistical and machine learning models, as well as (2) enhance climate science communication.

Existing climate indicator datasets often have limitations, such as a restricted number of indicators^7,8^, data limited to averages across climatological periods^10^, availability only at coarse spatial resolutions^11,12^, or a narrow focus^9^. This dataset seeks to overcome these gaps by providing high-resolution climate indicators that encompass many meteorological variables, multiple temporal aggregation windows, and climatological averages.

We aim to update the data repository annually once all source data are available, ensuring timely integration of the previous year to maintain the practical relevance of this dataset. We further intend to extend the workflow to future datasets, including the next generation of Austrian climate scenarios.

## Methods

### Input data

All climate indicators are derived from a range of existing datasets that provide comprehensive meteorological and hydrological variables. These datasets include daily records of minimum, average and maximum temperature^13^, precipitation totals^14^, sunshine duration^15^, snow-related variables (snow depth, snowmelt, total snow water equivalent and surface runoff)^16^ and drought-related variables (reference evapotranspiration and the derived Standardized Precipitation Evapotranspiration Index, SPEI)^17^.

All input data are available at a daily resolution, spanning the time period from 1961–2025. The dataset covers Austria and its surrounding catchments, extending beyond national borders, and is provided in the *ETRS89 / Austria Lambert* projected coordinate system (EPSG:3416). These datasets are openly available from the GeoSphere Austria Data Hub^18–20^. They are derived from quality-controlled input data, primarily observations from automatic weather stations, ensuring their reliability and consistency. For details on the quality of the underlying data, please refer to the original publications and their evaluation sections^13–17^.

The data were primarily used in their original form without additional pre-processing. This streamlined approach ensures compatibility and consistency across all input data sources.

### Calculation of climate indicators

The climate indicators are derived following established definitions and guidelines^21–24^ in order to cover a wide range of use cases. Detailed definitions for all climate indicators are provided in Appendix A. To ensure transparency and reproducibility, all routines required to replicate the calculation of climate indicators are open source (see the Code Availability section). The Python-based implementation includes boilerplate code for handling data input/output, managing metadata, and performing indicator calculations. These calculations are executed either by wrapping the xclim library^24^ or through custom-built routines. In some cases, pull requests have been contributed upstream to the main xclim repository. The code also supports data aggregation across different dimensions, and all steps in the computation processes are logged for traceability.

The calculation workflow is controlled via a configuration file, which specifies the technical metadata, such as input data paths. A pseudocode representation of the workflow is provided in Algorithm 1.

#### Algorithm 1

Pseudocode for the calculation of a climate indicator.

The main workflow for calculating climate indicators consists of the following steps:

1. **Load input data:** The appropriate input dataset is loaded based on the specific climate indicator being calculated. For precipitation data, metadata is corrected to accurately represent precipitation as a flux quantity (kg m^−2^ day^−1^), addressing inconsistencies in the original input data.
2. **Calculate thresholds (if required):** Some indicators rely on thresholds, which may be constant values or percentiles derived for a specific time period. Constant thresholds require no additional computation, while percentile-based thresholds are calculated once and stored separately to optimize computational efficiency. Detailed definitions of all climate indicators are provided in Appendix A.
3. **Calculate climate indicator:** Climate indicators are computed by either wrapping the xclim library^24^, if the required indicator is implemented, or custom-built routines for indicators not covered by xclim.
4. **Set attributes:** Output NetCDF data is stored with metadata attributes to ensure that the results are well-documented and self-descriptive. Metadata is structured in accordance with the CF Metadata Conventions, specifically adhering to CF-1.7 standards.
5. **Write output files:** The calculated climate indicators are saved as NetCDF files, organized by year. Each output file includes comprehensive metadata to facilitate data handling, interpretation and reuse.
6. **Aggregation and evaluation:** The computed climate indicators are aggregated and evaluated. Climatological reference periods are calculated by temporally averaging over 1961–1990 and 1991–2020, respectively, yielding maps of climatological averages. Additionally, a two-tailed Mann-Whitney *U* test is performed for all climate indicators to assess differences between the two climatological periods at a 95% confidence level, with the resulting *p*-values stored for further analysis. Furthermore, spatial averages are derived by computing the arithmetic mean across all grid cells, yielding time series for all climate indicators.

This workflow is repeated for all 117 climate indicators.

To ensure reproducibility and efficient workflow management, we leverage pytask as a workflow management system^25^. pytask creates a directed acyclic graph (DAG) to resolve task dependencies, automates the execution of the processing steps outlined above, and ensures that all calculations are reproducible and consistent. This approach minimizes manual intervention, streamlines the processing of large datasets, and enhances the overall transparency of the data analysis pipeline.

## Data Records

The dataset is publicly available via Zenodo^26^. It is organized into six zipped folders, containing the climate indicators along with various aggregations and derivations. Additionally, an image file (snow_yea_warming_stripes.png) is included as an example visualization, showcasing a small subset of the data in an aggregated form. The folders are structured hierarchically, with all six representing the top level of the directory tree. 

The hierarchical structure of the dataset comprises the following levels and corresponding folders:

•^1^**Root directory**: The top-level directory of the zipped folders (detailed further in the subsequent sections) includes the following folders:

– austrian_climate_indicators

– austrian_climate_indicators_areamean

– austrian_climate_indicators_clim_1991_2020

– austrian_climate_indicators_clim_1961_1990

– austrian_climate_indicators_significance

– austrian_climate_indicators_plots

• ^2^**group**: This level organizes the different input parameters for the climate indicators into groups. The subfolders influde: humidity, precipitation, mixed, radiation, runoff, snow, temperature, and thresholds. The thresholds subfolder contains pre-calculated thresholds required for specific climate indicators.

• ^3^**climate_indicator**: This hierarchical level is named according to the abbreviation of the respective climate indicator. A complete list of climate indicators and their abbreviations can be found in Appendix A.

• ^4^**aggregation_period**: This level specifies the temporal aggregation periods, which vary depending on the climate indicator. Most indicators are available with both annual (yea) and seasonal (sea) aggregations, but some are defined for hydrological periods (hydrological_year), or half-year periods (summer_halfyear, winter_halfyear).

• ^5^**netcdf_files**: This final level contains the data as NetCDF (.nc) files. Each file contains self-describing metadata.

This hierarchical structure was chosen to organize the dataset systematically and allow for straightforward navigation and access to the data. The contents and descriptions of the compressed folders are outlined in the following subsections.

### The austrian_climate_indicators.zip file

**Fig. 1:**
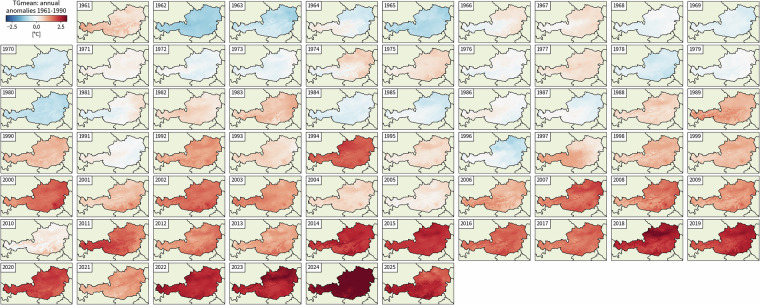
Stampplot of a climate indicator (TGmean) as anomaly relative to the reference period 1961–1990, demonstrating the full spatiotemporal dimensionality of the dataset.

**Fig. 2:**
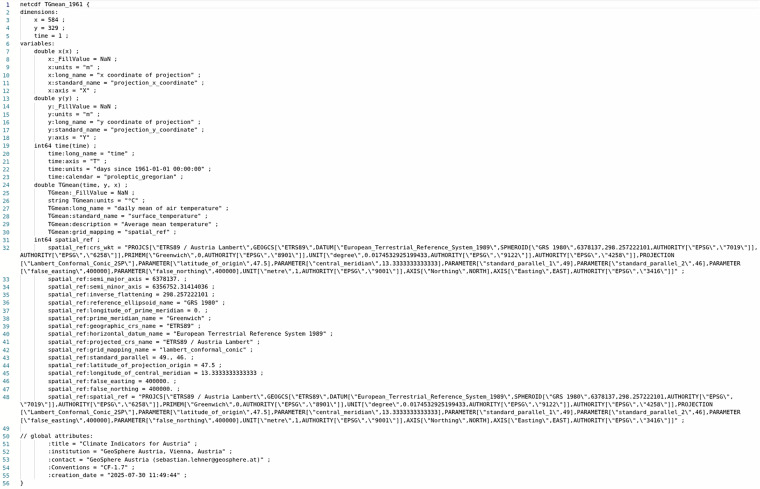
Example metadata for a climate indicator (TGmean) generated via ncdump -h.

This zipped folder contains the complete set of annual and seasonal climate indicators for the full spatial grid. It represents the main dataset for climate impact analyses and provides data at full spatiotemporal resolution, enabling detailed investigations of climate patterns and trends. Figure 1 illustrates an example of a climate indicator (mean annual daily average temperature, TGmean), showcasing the full spatiotemporal dimensionality of the data. Additionally, Fig. 2 presents an exemplary metadata structure for a TGmean output file for the year 1961.

### The austrian_climate_indicators_areamean.zip file

**Fig. 3:**
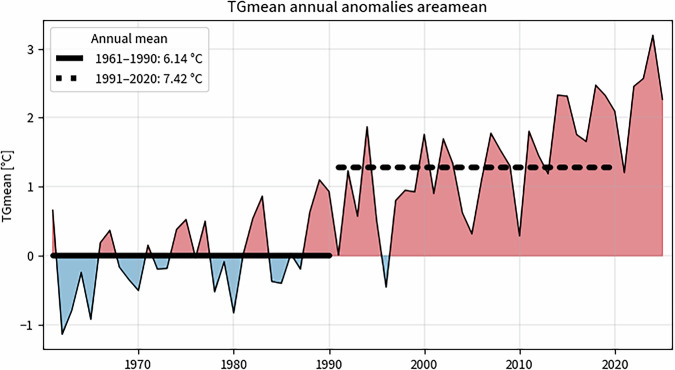
Spatially averaged anomalies of an annual climate indicator (TGmean). Anomalies are computed with respect to the 1961–1990 climatological average (solid line; by definition at 0, but included to visually emphasize the temporal reference period) and compared to the 1991–2020 climatological average (dashed line).

This folder contains spatially averaged time series for all climate indicators, providing an aggregated view of the data for national-scale analyses in time. Figure 3 shows an example of anomaly time series derived from the spatially averaged data, highlighting deviations from the 1961–1990 climatological average while providing a comparison with the most recent climatological standard normal 1991–2020.

### The austrian_climate_indicators_clim_1961_1990.zip file

**Fig. 4:**
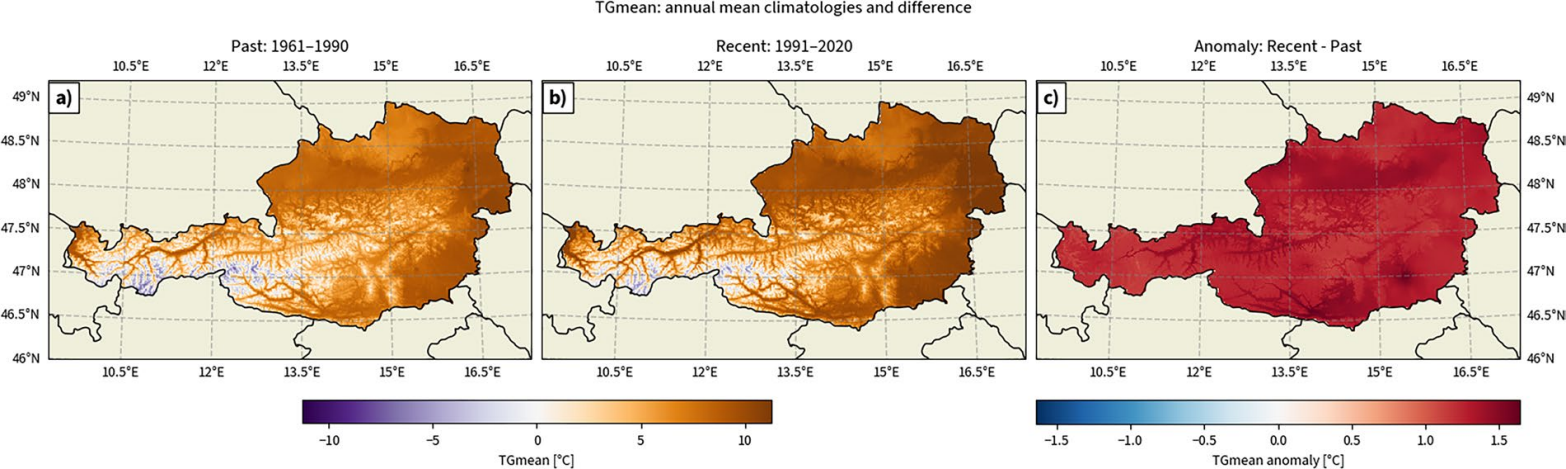
Aggregated fields for one climate indicator (TGmean) over two climatological reference periods: a) spatial average for 1961–1990 (past), b) spatial average for 1991–2020 (recent), and c) their difference (recent minus past).

This folder contains temporally averaged climate indicators for the 1961–1990 climatological standard normal. These aggregated data are provided for convenience, as this reference period is widely used in climate research. Figure 4 illustrates an example, comparing spatial averages for two climatological periods.

### The austrian_climate_indicators_clim_1991_2020.zip file

This folder contains temporally averaged climate indicators for the 1991–2020 climatological standard normal, similar to the previous section, but for the more recent period.

### The austrian_climate_indicators_significance.zip file

This folder contains results from significance tests between the two climatological periods (1961–1990 and 1991–2020). These data are provided to support analysis of statistically significant changes in climate indicators over time, which is a common use case in climate impact analyses.

### The austrian_climate_indicators_plots.zip file

This folder contains visualizations of the climate indicators, offering a convenient way to explore the data. The following types of plots are included:

• **Time series**: Spatially averaged anomalies relative to the 1961–1990 climatological reference period, with comparisons to the recent 1991–2020 average (Figure 3).

• **Spatial plots**: Single fields for climatologies and composite plots showing both climatologies and their differences (Figure 4).

• **Stampplots**: Visualizations of the full spatiotemporal dimensionality of the data (Figure 1).

• **Grouped significance plots**: Portions of significant changes within a parameter group (e.g., temperature, precipitation) across periods (not shown).

• **Composite warming stripes**: Standardized anomalies for all climate indicators grouped by parameter category (not shown).

## Technical Validation

The technical validation of the generated dataset was conducted through a series of checks to ensure data integrity, consistency, and plausibility. Specifically, the following steps were performed:

• **File size check**: All generated files were verified to exceed a predefined size threshold to capture erroneously produced files.

• **File count validation**: The number of output files per climate indicator was checked against the expected count, which is determined by the configured start and end year (as each year is saved in a separate file).

• **Value verification**: Files were checked to ensure that they contain valid values and are not populated solely with missing values (NaNs).

The results of these technical validation checks are logged to a file to provide a detailed record for identifying and addressing any errors.

In addition, all generated figures were manually reviewed to confirm the plausibility and quality of the curated data. This step ensures that the visual representations align with expected climate patterns and trends from a climatological perspective.

The source data itself adheres to internal quality standards established by the Austrian national service for geology, geophysics, climatology, and meteorology (GeoSphere Austria), ensuring validity and reliability of the input data.

Concerning software, the Python libraries used in the workflow incorporate robust quality control mechanisms, including testing, thorough code review and internal metadata checks. These checks enforce conventions, proper units and other relevant standards (see^24^ for details). Together, these measures provide a high level of quality assurance for both the dataset and the underlying computational processes, thereby upholding stringent standards and conventions that are essential to foster trust in reliable climate research.

## Usage Notes

The climate indicators provided in this dataset are well-suited for studies investigating changes, trends, or dependencies in related climate impact phenomena. These encompass a wide range of areas, including natural hazards^27^, forestry^28^, public health^29^, socio-ecologic systems, and economic impacts such as crop yields^30,31^. For comprehensive impact analyses, it is essential to incorporate additional risk drivers beyond climate variables to ensure a holistic characterization of impacts and associated risks^27,32^. Furthermore, the choice of reference periods for all applications should be carefully considered and thoroughly documented. Comparing indicators based on different reference periods can cause unexpected and diverging results, in particular for percentile-based extreme indices^33^. Therefore, indicators that rely on a reference period for threshold calculation should, whenever possible, be compared based on the same reference period. Note that no homogenization procedure is applied within the presented workflow. However, the input data are derived from a consistent set of quality-controlled station data. Nevertheless, trend analyses should account for possible discrepancies compared to fully homogenized time series.

The dataset can also be used to train climate impact models, offering a robust foundation for exploring the relationships between climate indicators and various impact metrics.

Users of this dataset should be mindful of multicollinearity, which is a common characteristic of meteorological variables and, by extension, climate indicators derived from the same input data. When working with multiple climate indicators, it is essential to carefully assess correlations between them and employ appropriate feature selection techniques. These techniques should be guided by the underlying research question, with careful consideration of the balance between predictive accuracy and explanatory aims^34^. Neglecting to address this issue can lead to issues such as reduced model performance and biased inference (e.g. in feature importance assessments) within statistical learning models^35,36^. Spatial multicollinearities among the climate indicators in this dataset have been analyzed and discussed in^6^, providing additional insights for users seeking to incorporate these data in their analyses.

## Supplementary information

Supplementary information

## Data Availability

All calculated climate indicators, spatiotemporal aggregations, and generated figures are publicly available via Zenodo at 10.5281/zenodo.16928609. The source data used to calculate all climate indicators can be accessed through the GeoSphere Austria DataHub, specifically the gridded datasets (a) SPARTACUS (10.60669/m6w8-s545), (b) WINFORE (10.60669/f6ed-2p24), and (c) SNOWGRID (10.60669/fsxx-6977).

## Code availability

All code for calculating climate indicators, performing aggregations and significance tests, as well as generating visualizations, is open source and available on GitHub at https://github.com/seblehner/austrian-climate-indicators.
