# Supplementary material for: Climate indicators for Austria since 1961 at 1 km resolution
Source: Sci Data. 2026 Feb 18;13:475. doi: 10.1038/s41597-026-06834-y (PMC13031643; doi:10.1038/s41597-026-06834-y)
Supplement: Supplementary file 1 — Supplementary information [file 41597_2026_6834_MOESM1_ESM.pdf]

Supplement to: Climate indicators for Austria since 1961 at 1 km resolution

Sebastian Lehner[1,2,*] and Matthias Schlögl[1,3]

[1]Department for Climate Impact Research, GeoSphere Austria, Hohe Warte 38, Vienna, 1190, Austria
[2]Department of Meteorology and Geophysics, University of Vienna, Josef-Holaubek-Platz 2, Vienna, 1090, Austria
[3]Institute of Mountain Risk Engineering, BOKU University, Peter-Jordan Straße 82, Vienna, 1190, Austria
[*]Corresponding author: sebastian.lehner@geosphere.at

Appendix A Definitions of climate indicators

This section provides definitions for all climate indicators. Here, x represents the data points (parameter), t corresponds to a daily timesteps for all days within the respective time period (annual or seasonal, etc.), and n denotes the number of days within the same time period. To simplify the presentation, all climate indicators are calculated on a per-gridcell basis, hence the spatial dimension is excluded in the definitions.

Temperature-based climate indicators

The input variables used are `TX` for daily maximum temperature, `TN` for daily minimum temperature and `TG` for daily average temperature, all in degrees Celsius.

`TGmean`: The average daily mean temperature, calculated as:

$$f(\mathrm{TG}) = \frac{1}{n}\sum_{t=1}^{n} \mathrm{TG}_t \tag{1}$$

`TNmean`: The average daily minimum temperature, calculated as:

$$f(\mathrm{TN}) = \frac{1}{n}\sum_{t=1}^{n} \mathrm{TN}_t \tag{2}$$

`TXmean`: The average daily maximum temperature, calculated as:

$$f(\mathrm{TX}) = \frac{1}{n}\sum_{t=1}^{n} \mathrm{TX}_t \tag{3}$$

`FD`: The number of frost days, calculated as:

$$f(\mathrm{TN}) = \sum_{t=1}^{n} \begin{cases} 1, & \text{if } \mathrm{TN}_t < 0 \\ 0, & \text{otherwise.} \end{cases} \tag{4}$$

`SU`: The number of summer days, calculated as:

$$f(\mathrm{TX}) = \sum_{t=1}^{n} \begin{cases} 1, & \text{if } \mathrm{TX}_t > 25 \\ 0, & \text{otherwise.} \end{cases} \tag{5}$$

`HD`: The number of hot days, calculated as:

$$f(\mathrm{TX}) = \sum_{t=1}^{n} \begin{cases} 1, & \text{if } \mathrm{TX}_t > 30 \\ 0, & \text{otherwise.} \end{cases} \tag{6}$$

`ID`: The number of icing days, calculated as:

$$f(\mathrm{TX}) = \sum_{t=1}^{n} \begin{cases} 1, & \text{if } \mathrm{TX}_t < 0 \\ 0, & \text{otherwise.} \end{cases} \tag{7}$$

`TR`: The number of tropical nights, calculated as:

$$f(\mathrm{TN}) = \sum_{t=1}^{n} \begin{cases} 1, & \text{if } \mathrm{TN}_t > 20 \\ 0, & \text{otherwise.} \end{cases} \tag{8}$$

`KYS`: Kysely days are defined as the count of days in periods of at least 3 consecutive days where (1) TX $\geq$ 30 °C on the first day, (2) TX $\geq$ 25 °C on all subsequent days, and (3) and the average maximum temperature across all days in the period fulfills TX $\geq$ 30 °C.

`GSS`: The day of the year corresponding to the first day of a four-day period during which TG $>$ 5 °C on all days in the window.

`GSL`: The number of days between the start and end of the growing season. The growing season begins with a 6-day period where TG $>$ 5 °C on all days in the window and ends with a 6-day period where TG $<$ 5 °C on all days.

`spring_backlash`: The number of events counted as days where $\text{TN}_t < -2$ °C for all days t after `GSS` and up to day 180 of the year. This is associated with spring frost.

`SBI`: The spring backlash index represents the growing degree days above 5 °C (see `GDD5`) for all days t occurring after `GSS` and up to the last spring backlash event.

`TXx`: The maximum daily maximum temperature, calculated as:

$$f(\text{TX}) = \max(\text{TX}) \tag{9}$$

`TNx`: The maximum daily minimum temperature, calculated as:

$$f(\text{TN}) = \max(\text{TN}) \tag{10}$$

`TXn`: The minimum daily maximum temperature, calculated as:

$$f(\text{TX}) = \min(\text{TX}) \tag{11}$$

`TNn`: The minimum daily minimum temperature, calculated as:

$$f(\text{TN}) = \min(\text{TN}) \tag{12}$$

`TN10p`: This indicator represents the percentage of days where the daily

minimum temperature (TN_t) is below the 10th percentile (TN_{10p}) of the reference period (1991–2020). It is calculated as:

$$f(\mathrm{TN}) = \frac{100[\%]}{n_d} \sum_{t=1}^{n} \begin{cases} 1, & \text{if } \mathrm{TN}_t < \mathrm{TN}_{10p} \\ 0, & \text{otherwise.} \end{cases} \tag{13}$$

where n_d is the number of days in the period under consideration (e.g. annual or seasonal). For the annual aggregation, TN_{10p} is derived from all data in the reference period. For seasonal aggregation, TN_{10p} is calculated using data specific to each season within the reference period.

`TX10p`: Defined similarly to Equation 13, but with TX instead of TN:

$$f(\mathrm{TX}) = \frac{100[\%]}{n_d} \sum_{t=1}^{n} \begin{cases} 1, & \text{if } \mathrm{TX}_t < \mathrm{TX}_{10p} \\ 0, & \text{otherwise.} \end{cases} \tag{14}$$

`TN90p`: Defined similarly to Equation 13, but with $\mathrm{TN}_t > \mathrm{TN}_{90p}$:

$$f(\mathrm{TN}) = \frac{100[\%]}{n_d} \sum_{t=1}^{n} \begin{cases} 1, & \text{if } \mathrm{TN}_t > \mathrm{TN}_{90p} \\ 0, & \text{otherwise.} \end{cases} \tag{15}$$

`TX90p`: Defined similarly to Equation 13, but for TX instead of of TN and $\mathrm{TX}_t > \mathrm{TX}_{90p}$:

$$f(\mathrm{TX}) = \frac{100[\%]}{n_d} \sum_{t=1}^{n} \begin{cases} 1, & \text{if } \mathrm{TX}_t > \mathrm{TX}_{90p} \\ 0, & \text{otherwise.} \end{cases} \tag{16}$$

`CFD`: Consecutive frost days, defined as the maximum length of consecutive periods where $\mathrm{TN} < 0$ °C.

`CSU`: Consecutive summer days, defined as the maximum length of consecutive periods where $\mathrm{TX} > 25$ °C.

`WSDI`: The warm spell duration index reflects the number of days within warm spells. Warm spells are defined as consecutive periods of at least 6 days where $\mathrm{TX} > \mathrm{TX}_{90p}$. Here, TX_{90p} is the 90th percentile of TX based on the reference period 1991–2020.

`CSDI`: The cold spell duration index reflects the number of days within cold spells. Cold spells are defined as consecutive periods of at least 6 days where $\mathrm{TN} < \mathrm{TN}_{10p}$. Here, TN_{10p} is the 10th percentile of TN based on the reference period 1991–2020.

`HSF`: The hot spell frequency denotes the number of hot spells as defined in the `WSDI` indicator.

`CSF`: The cold spell frequency denotes the number of cold spells as defined as in the `CSDI` indicator.

`CE`: Cold episodes reflect the number of cold spells, defined as continuous periods of at least 5 days where $\mathrm{TX} < 0$ °C.

`EID`: The number of days where $\mathrm{TN} < -7$ °C and $\mathrm{TX} < 0$ °C.

`TPD`: The number of days where $\mathrm{TG} > 1.5$ °C.

`FTD`: The number of days where $\mathrm{TN} < 0$ °C and $\mathrm{TX} > 0$ °C.

`FTD2`: The number of days where $\mathrm{TN} < -2$ °C and $\mathrm{TX} > 2$ °C.

`continentality`: The difference between the maximum and minimum average monthly temperature:

$$f(\mathrm{TG}) = \max(\mathrm{TG}_{mon}) - \min(\mathrm{TG}_{mon}) \tag{17}$$

where TG_{mon} represents the average monthly TG.

`GDD0`: The growing degree days, defined as the sum of daily temperature differences above a threshold of 0 °C:

$$f(\mathrm{TG}) = \sum_{t=1}^{n} \begin{cases} \mathrm{TG}_t - c, & \text{if } \mathrm{TG}_t > c \\ 0, & \text{otherwise.} \end{cases} \tag{18}$$

where c is a constant with value 0 °C.

`GDD5`: Similar to Equation 18 but with a threshold of $c = 5$ °C.

`GDD10`: Similar to Equation 18 but with a threshold of $c = 10$ °C.

DTR: The average daily temperature range, calculated as:

$$f(\mathrm{TX}, \mathrm{TN}) = \frac{1}{n_d} \sum_{t=1}^{n} \mathrm{TX}_t - \mathrm{TN}_t \tag{19}$$

where n_d is the number of days in the period under consideration (e.g. annual or seasonal).

ETR: The extreme temperature range is defined as the difference between the maximum daily maximum temperature (TXx) and the minimum daily minimum temperature (TNn):

$$f(\mathrm{TX}, \mathrm{TN}) = \mathrm{TX}x - \mathrm{TN}n \tag{20}$$

BIO01: First bioclimatic indicator, representing the mean temperature calculated specifically for an annual aggregation period. It is equivalent to the general mean temperature as defined in Equation 1.

BIO02: The mean diurnal temperature range is calculated as the average difference between daily maximum temperature (TX_t) and daily minimum temperature (TN_t):

$$f(\mathrm{TX}, \mathrm{TN}) = \frac{1}{n} \sum_{t=1}^{n} (\mathrm{TX}_t - \mathrm{TN}_t) \tag{21}$$

BIO03: The ratio of the mean diurnal temperature range (BIO02) and the annual temperature range (BIO07):

$$f(\mathrm{TX}, \mathrm{TN}) = \frac{\mathrm{BIO02}}{\mathrm{BIO07}} \tag{22}$$

BIO04: Temperature seasonality is defined as the annual coefficient of variation in temperature, expressed as percentage:

$$f(\mathrm{TG}) = 100[\%] \cdot \frac{\sigma_{\mathrm{TG}}}{\mu_{\mathrm{TG}}} \tag{23}$$

where σ_{TG} is the standard deviation daily mean temperature (TG) per year, and μ_{TG} the mean of TG for the same year.

BIO05: The maximum daily maximum temperature (see Equation 9) aligns with the ANUCLIM definitions[1] as implemented in `xclim` but differs from the Bioclim definition, which refers to the maximum temperature *of the warmest month*.

BIO06: The maximum daily maximum temperature (see Equation 12) aligns with the ANUCLIM definitions[1] as implemented in `xclim` but differs from the Bioclim definition, which refers to the minimum temperature *of the coldest month*.

BIO07: The difference between the maximum daily maximum temperature (TXx) and the minimum daily minimum temperature (TNn). This is equivalent to Equation 20 for an annual aggregation period.

BIO10: The mean temperature for the warmest quarter, calculated as:

$$f(\mathrm{TG}) = \frac{1}{n}\sum_{j=1}^{n}\mathrm{TG}_j \tag{24}$$

where j is limited to the days within the season where

$$\text{season} = \arg\max(\mathrm{TG}_{sea}) \tag{25}$$

with TG_{sea} denoting the seasonal average temperature.

BIO11: The mean temperature for the coldest quarter, calculated as:

$$f(\mathrm{TG}) = \frac{1}{n}\sum_{j=1}^{n}\mathrm{TG}_j \tag{26}$$

where j is limited to the days within the season where

$$\text{season} = \arg\min(\mathrm{TG}_{sea}) \tag{27}$$

with TG_{sea} denoting the seasonal average temperature.

[1] Xu, T. & Hutchinson, M. F. New developments and applications in the ANUCLIM spatial climatic and bioclimatic modelling package. *Environmental Modelling & Software* **40**, 267–279, https://doi.org/10.1016/j.envsoft.2012.10.003 (2013).

`CDDcold18.3`: The sum of daily temperature differences above a threshold of 18.3 °C, calculated as:

$$f(\mathrm{TG}) = \sum_{t=1}^{n} \begin{cases} \mathrm{TG}_t - c, & \text{if } \mathrm{TG}_t > c \\ 0, & \text{otherwise.} \end{cases} \tag{28}$$

where c is a constant with value 18.3 °C.

`HDDheat20`: The sum of daily temperature differences below a threshold of 20 °C, calculated as:

$$f(\mathrm{TG}) = \sum_{t=1}^{n} \begin{cases} c - \mathrm{TG}_t, & \text{if } \mathrm{TG}_t < c \\ 0, & \text{otherwise.} \end{cases} \tag{29}$$

where c is a constant with value 20 °C.

Precipitation-based climate indicators

The input variable used is `RR` for daily precipitation totals in mm (corresponding to $\mathrm{kg\,m^{-2}\,day^{-1}}$). Wet days are defined as days where RR > 1 mm.

`PRCPTOT`: The total precipitation accumulated over a specified period, calculated as:

$$f(\mathrm{RR}) = \sum_{t=1}^{n} \mathrm{RR}_t \tag{30}$$

`Rx1day`: The maximum daily precipitation within a specified period, calculated as:

$$f(\mathrm{RR}) = \max(\mathrm{RR}_t) \tag{31}$$

`Rx5day`: The maximum 5-day precipitation within a specified period, calculated as:

$$f(\mathrm{RR}) = \max(\mathrm{RR}_{5t}) \tag{32}$$

where RR_{5t} represents the total precipitation accumulated within a 5-day rolling window.

`RR90pct`: The 90th percentile of daily precipitation totals for wet days ($\mathrm{RR}_{wet,90p}$) within a specified period:

$$f(\mathrm{RR}) = \mathrm{RR}_{wet,90p}) \tag{33}$$

`RR95pct`: Similar to Equation 33 but for the 95th percentile.

`RRmean`: The average daily precipitation within a specified period, calculated as:

$$f(\mathrm{RR}) = \frac{1}{n} \sum_{t=1}^{n} \mathrm{RR}_t \tag{34}$$

`SDII`: The simple daily precipitation intensity index is defined as the average precipitation intensity on wet days, calculated as:

$$f(\mathrm{RR}) = \frac{1}{n_{wet}} \sum_{t=1}^{n_{\mathrm{wet}}} \mathrm{RR}_{t,wet} \tag{35}$$

where n_{wet} is the number of wet days in the period under consideration, and $\mathrm{RR}_{t,wet}$ represents the precipitation totals on wet days.

`R10mm`: The count of moderate wet days, calculated as:

$$f(\mathrm{RR}) = \sum_{t=1}^{n} \begin{cases} 1, & \text{if } \mathrm{RR}_t > c \\ 0, & \text{otherwise.} \end{cases} \tag{36}$$

where $c = 10$ mm.

`R20mm`: Similar to Equation 36 but with $c = 20$ mm.

`R30mm`: Similar to Equation 36 but with $c = 30$ mm.

`R40mm`: Similar to Equation 36 but with $c = 40$ mm.

`R50mm`: Similar to Equation 36 but with $c = 50$ mm.

`CDD`: Consecutive dry days, defined as the number of days in the longest consecutive period where daily precipitation is less than 1 mm.

`CWD`: Consecutive wet days, defined as the number of days in the longest consecutive period where daily precipitation exceeds 1 mm.

`Rsum30min`: The minimum accumulated precipitation within any 30-day rolling window over a specified period, calculated as:

$$f(\mathrm{RR}) = \min(\sum_{t}^{n} \mathrm{RR}_{30t}) \tag{37}$$

where RR_{30t} denotes the precipitation totals accumulated within a rolling 30-day window.

`Rsum30max`: The maximum accumulated precipitation over any rolling 30-day window, calculated as:

$$f(\mathrm{RR}) = \max(\sum_{t}^{n} \mathrm{RR}_{30t}) \tag{38}$$

where RR_{30t} represents the total precipitation accumulated within a 30-day rolling window.

`R75p`: The total precipitation accumulated on moderately wet days within a specified period, which are defined as days with daily precipitation exceeding the 75th percentile of wet day precipitation during the reference period (1991–2020). The indicator is calculated as:

$$f(\mathrm{RR}) = \sum_{t=1}^{n} \begin{cases} \mathrm{RR}_t, & \text{if } \mathrm{RR}_t > \mathrm{RR}_{wet,75p} \\ 0, & \text{otherwise.} \end{cases} \tag{39}$$

where $\mathrm{RR}_{wet,75p}$ is the 75th percentile of RR_{wet} for all wet days during the reference period 1991–2020.

`R90p`: Similar to Equation 39, but for the 90th percentile.

`R95p`: Similar to Equation 39, but for the 95th percentile.

`R99p`: Similar to Equation 39, but for the 99th percentile.

R90pTOT: The percentage contribution of precipitation totals from RR $>$ $\mathrm{RR}_{wet,90p}$ within a specified period, calculated as:

$$f(\mathrm{RR}) = \frac{100[\%]}{\sum_{t=1}^{n} \mathrm{RR}_t} \sum_{t=1}^{n} \begin{cases} \mathrm{RR}_t, & \text{if } \mathrm{RR}_t > \mathrm{RR}_{wet,90p} \\ 0, & \text{otherwise.} \end{cases} \tag{40}$$

where $\mathrm{RR}_{wet,90p}$ is the 90th percentile of RR_{wet} for all wet days during the reference period 1991–2020.

R95pTOT: Similar to Equation 40, but for the 95th percentile.

R99pTOT: Similar to Equation 40, but for the 99th percentile.

API07_Q95: This temporally aggregated version of the antecedent precipitation index (API) represents the 95th percentile of a weighted sum of daily precipitation totals over a specified time window with a length of k days and a weighting exponent p. The basic API is calculated as:

$$f(\mathrm{RR}_t) = \sum_{i=t}^{t-k} \mathrm{RR}_i \cdot p^i \tag{41}$$

where $k = 7$ and $p = 0.935$. This indicator is the 95th percentile of that.

API14_Q95: Similar to Equation 41, but with $k = 14$.

API28_Q95: Similar to Equation 41, but with $k = 28$.

PCI_Q95: This temporally aggregated version of the precipitation concentration index (PCI) represents the 95th percentile of the PCI, which quantifies the concentration of monthly precipitation totals within a given period. The PCI is defined as follows:

$$f(\mathrm{RR}) = \frac{\sum_j \left(\mathrm{RR}_{mon}^2\right)}{\left(\sum_{i=1}^{n} \mathrm{RR}_t\right)^2} \tag{42}$$

where RR_{mon} denotes the monthly accumulated precipitation totals, and j represents all months in the specified period (e.g. months within a season or year).

BIO12: This indicator represents the total annual precipitation, calculated as the sum of daily precipitation totals over an entire year. It is equivalent to Equation 30, with the aggregation specifically applied to an annual time scale.

BIO13: The maximum daily precipitation within a year:

$$f(\mathrm{RR}) = \max \mathrm{RR}_t \tag{43}$$

BIO14: The minimum daily precipitation within a year:

$$f(\mathrm{RR}) = \min \mathrm{RR}_t \tag{44}$$

BIO15: The annual coefficient of variation of precipitation, expressed as a percentage:

$$f(\mathrm{RR}) = 100[\%] \cdot \frac{\sigma_{\mathrm{RR}}}{\mu_{\mathrm{RR}}} \tag{45}$$

where σ_{RR} is the standard deviation of RR per year, and μ_{RR} the mean of RR for the same year.

BIO16: The total precipitation during the wettest season of the year, calculated as:

$$f(\mathrm{RR}) = \sum_{j=1}^{n} \mathrm{RR}_j \tag{46}$$

where j is limited to the days within the season where

$$\mathrm{season} = \arg\max(\mathrm{RR}_{sea}) \tag{47}$$

with RR_{sea} denoting the total accumulated precipitation for each season.

BIO17:

$$f(\mathrm{RR}) = \sum_{j=1}^{n} \mathrm{RR}_j \tag{48}$$

where j is limited to the days inside the season where

$$\mathrm{season} = \arg\min(\mathrm{RR}_{sea}) \tag{49}$$

where RR_{sea} is the seasonal total accumulated precipitation.

`RR_summerhalf`:

$$f(\mathrm{RR}) = \sum_{j=1}^{n} \mathrm{RR}_j \tag{50}$$

where j is limited to the days within the months April to September.

`RR_winterhalf`:

$$f(\mathrm{RR}) = \sum_{j=1}^{n} \mathrm{RR}_j \tag{51}$$

where j is limited to the days within the months October to March.

Radiation-based climate indicators

These indicators utilize sunshine duration as input variables to quantify solar radiation. Specifically, the two key input variables used are `SA` for daily absolute sunshine duration in hours, and `SR` for daily relative sunshine duration, expressed as a fraction.

`SAmean`: The mean daily absolute sunshine duration over a specified period:

$$f(\mathrm{SA}) = \frac{1}{n} \sum_{t=1}^{n} \mathrm{SA}_t \tag{52}$$

`SRmean`: The mean daily relative sunshine duration over a specified period:

$$f(\mathrm{SR}) = \frac{1}{n} \sum_{t=1}^{n} \mathrm{SR}_t \tag{53}$$

Humidity-based climate indicators

The input variables used are ET0 for daily reference evapotranspiration in mm, and SPEI as the Standardized Precipitation Evaporation Index[2] for 30, 90 and 365 days (SPEI30, SPEI90 and SPEI365), respectively.

ET0mean: The the average daily reference evapotranspiration over a specified period, calculated as:

$$f(\mathrm{ET0}) = \frac{1}{n}\sum_{t=1}^{n} \mathrm{ET0}_t \tag{54}$$

ET0_seasonality: The seasonality of ET0 quantifies the variability of reference evapotranspiration across months within a specified period. It is calculated in two steps:

$$f(\mathrm{ET0}_{mon}) = \sqrt{\frac{1}{n_{mon}}\sum_{t=1}^{n_{mon}} \left(\mathrm{ET0}_t - \overline{\mathrm{ET0}_{mon}}\right)^2} = \sigma_{\mathrm{ET0},mon} \tag{55}$$

where $\overline{\mathrm{ET0}_{mon}}$ is the monthly mean, and $\sigma_{\mathrm{ET0},mon}$ is the monthly standard deviation of ET0.
The final indicator is derived by averaging the monthly standard deviations over all months in the aggregation period:

$$f(\mathrm{ET0}) = \frac{1}{n_{months}}\sum_{j=1}^{n_{months}} \sigma_{\mathrm{ET0},j} \tag{56}$$

SPEI30mean: The average value of the SPEI calculated over a 30-day period within a specified time frame:

$$f(\mathrm{SPEI30}) = \frac{1}{n}\sum_{t=1}^{n} \mathrm{SPEI30}_t \tag{57}$$

SPEI90mean: Similar to Equation 57, but with SPEI90 instead of SPEI30.

[2]Vicente-Serrano, S. M., Beguería, S. & López-Moreno, J. I. A Multiscalar Drought Index Sensitive to Global Warming: The Standardized Precipitation Evapotranspiration Index. *Journal of Climate* **23**, 1696–1718, https://doi.org/10.1175/2009JCLI2909.1 (2010).

SPEI365mean: Similar to Equation 57, but with SPEI365 instead of SPEI30.

SPEI90_th-2: The count of days within a specified period where the SPEI for a 90-day window falls below a threshold of −2:

$$f(\text{SPEI90}) = \sum_{t=1}^{n} \begin{cases} 1, & \text{if SPEI90} < c \\ 0, & \text{otherwise.} \end{cases} \tag{58}$$

where $c = -2$ mm.

SPEI90_th-1: Similar to Equation 58, but with $c = -1$.

SPEI90_th+1: The count of days within a specified period where the SPEI for a 90-day window exceeds a threshold of 1:

$$f(\text{SPEI90}) = \sum_{t=1}^{n} \begin{cases} 1, & \text{if SPEI90} > c \\ 0, & \text{otherwise.} \end{cases} \tag{59}$$

where $c = 1$ mm.

SPEI90_th+2: Similar to Equation 59, but with $c = 2$.

Snow-based climate indicators

The input variables are:

- SDE - daily total height of snow (snow depth) in m,
- SF - daily amount of fresh snow (snowfall) in m,
- SWE_tot - daily total snow water equivalent in $\text{kg}\,\text{m}^{-2}$,
- SWE_fr - daily fresh amount of available total snow water equivalent in $\text{kg}\,\text{m}^{-2}$,
- snowmelt - daily amount of melted snow in kgm^{-2},
- runoff - daily total surface runoff from rain and snowmelt in m.

HS_Q95: The 95th percentile of SDE.

HSfr_Q95: The 95th percentile of SF.

SWE_Q95: The 95th percentile of SWE_tot.

SWEfr_Q95: The 95th percentile of SWE_fr.

NSD_72h: The maximum 3-day fresh snow depth ('new snow depth within 72 hours') within a specified period, calculated as:

$$f(\mathrm{SF}) = \max(\mathrm{SF}_{3t}) \tag{60}$$

where SF_{3t} denotes the 3-day SF, calculated using a 3-day running window.

SCD01: The count of days within a specified period where the daily snow depth exceeds 1 cm, calculated as:

$$f(\mathrm{SDE}) = \sum_{t=1}^{n} \begin{cases} 1, & \text{if SDE} > c \\ 0, & \text{otherwise.} \end{cases} \tag{61}$$

where $c = 1$ cm.

SCD10: Similar to Equation 61, but with $c = 10$.

runoff_Q95: The 95th percentile of runoff.

snowmelt_Q95: The 95th percentile of snowmelt.

HN1_SD: The count of days within a specified period where the daily fresh snow depth exceeds 1 cm, calculated as:

$$f(\mathrm{SF}) = \sum_{t=1}^{n} \begin{cases} 1, & \text{if SF} > c \\ 0, & \text{otherwise.} \end{cases} \tag{62}$$

where $c = 1$ cm.

HN5_SD: Similar to Equation 62, but with $c = 5$ cm.

HN10_SD: Similar to Equation 62, but with $c = 10$ cm.

HN20_SD: Similar to Equation 62, but with $c = 20$ cm.

Climate indicators based on multiple input variables

`SHMI`: The summer heat moisture index is defined as the ratio of the maximum monthly average temperature (TG_{mon}) to the mean precipitation during summer months (June, July, August; RR_{JJA}), calculated as:

$$f(\mathrm{TG}, \mathrm{RR}) = \frac{\max(\mathrm{TG}_{mon})}{\mathrm{RR}_{JJA}} \tag{63}$$

`ET0_Qcold`: The mean reference evapotranspiration in the coldest quarter, calculated as:

$$f(\mathrm{ET0}) = \frac{1}{n}\sum_{j=1}^{n} \mathrm{ET0}_j \tag{64}$$

where j is limited to the days within the season where

$$\mathrm{season} = \arg\min(\mathrm{TG}_{sea}) \tag{65}$$

with TG_{sea} denoting the seasonal average temperature.

`ET0_Qdry`: The mean reference evapotranspiration in the driest quarter, calculated as:

$$f(\mathrm{ET0}) = \frac{1}{n}\sum_{j=1}^{n} \mathrm{ET0}_j \tag{66}$$

where j is limited to the days within the season where

$$\mathrm{season} = \arg\min(\mathrm{RR}_{sea}) \tag{67}$$

with RR_{sea} denoting the seasonal total accumulated precipitation.

`ET0_Qwarm`: The mean reference evapotranspiration in the warmest quarter, calculated as:

$$f(\mathrm{ET0}) = \frac{1}{n}\sum_{j=1}^{n} \mathrm{ET0}_j \tag{68}$$

where j is limited to the days within the season where

$$\mathrm{season} = \arg\max(\mathrm{TG}_{sea}) \tag{69}$$

with TG_{sea} denoting the seasonal average temperature.

ETO_Qwet: The mean reference evapotranspiration in the wettest quarter, calculated as:

$$f(\text{ET0}) = \frac{1}{n}\sum_{j=1}^{n} \text{ET0}_j \tag{70}$$

where j is limited to the days within the season where

$$\text{season} = \arg\max(\text{RR}_{sea}) \tag{71}$$

with RR_{sea} denoting the seasonal total accumulated precipitation.

BIO08: The mean temperature of the wettest quarter, calculated as:

$$f(\text{TG}) = \frac{1}{n}\sum_{j=1}^{n} \text{TG}_j \tag{72}$$

where j is limited to the days within the season where

$$\text{season} = \arg\max(\text{RR}_{sea}) \tag{73}$$

with RR_{sea} denoting the seasonal total accumulated precipitation.

BIO09: The mean temperature of the driest quarter, calculated as:

$$f(\text{TG}) = \frac{1}{n}\sum_{j=1}^{n} \text{TG}_j \tag{74}$$

where j is limited to the days within the season where

$$\text{season} = \arg\min(\text{RR}_{sea}) \tag{75}$$

with RR_{sea} denoting the seasonal total accumulated precipitation.

BIO18: The total precipitation during the warmest quarter, calculated as:

$$f(\text{RR}) = \sum_{j=1}^{n} \text{RR}_j \tag{76}$$

where j is limited to the days within the season where

$$\text{season} = \arg\max(\text{TG}_{sea}) \tag{77}$$

with TG_{sea} denoting the seasonal average temperature.

BIO19: The total precipitation during the coldest quarter, calculated as:

$$f(\mathrm{RR}) = \sum_{j=1}^{n} \mathrm{RR}_j \tag{78}$$

where j is limited to the days within the season where

$$\text{season} = \arg\min(\mathrm{TG}_{sea}) \tag{79}$$

with TG_{sea} denoting the seasonal average temperature.
